# Feasibility of Training Community Health Workers in the Detection of Oral Cancer

**DOI:** 10.1001/jamanetworkopen.2021.44022

**Published:** 2022-01-18

**Authors:** Vipin Thampi, Roopa Hariprasad, Amrita John, Suzanne Nethan, Kavitha Dhanasekaran, Vipin Kumar, Praveen Birur, J. S. Thakur, Richard Lilford, Nasir M. Rajpoot, Paramjit Gill

**Affiliations:** 1 Piramal Swasthya Research Institute, Hyderabad, India; 2 Indian Council of Medical Research, National Institute of Cancer Prevention and Research, Noida, India; 3 Shore Christian Fellowship, Rajnandgaon Chhatisgarh, India; 4 School of Preventive Oncology, Bihar, India; 5 Indian Council of Medical Research, National Institute of Cancer Prevention and Research, Noida, India; 6 E-Government Cell, Indian Council of Medical Research, New Delhi, India; 7 Oral Medicine and Radiology, KLE Institute of Dental Sciences and Lead-Oral Cancer Screening-Biocon Foundation, Bengaluru, India; 8 Department of Community Medicine & School of Public Health, Post Graduate Institute of Medical Education and Research, Chandigarh, India; 9 National Institute for Health Research Applied Research Collaboration West Midlands, University of Birmingham, Edgbaston, United Kingdom; 10 Department of Computer Science, Tissue Image Analytics Centre, University of Warwick, Coventry, United Kingdom; 11 Division of Health Sciences, University of Warwick, Coventry, United Kingdom

## Abstract

**Importance:**

Visual screening for oral cancer has been found to be useful in a large randomized clinical trial in Kerala, India, showing substantial reduction in mortality. To address the shortage of medical personnel in resource-deficient regions, using the services of community health workers has been proposed as a strategy to fill the gap in human resources in health care.

**Objective:**

To assess the feasibility of community health workers in screening and early detection of oral cancer using a mobile application capturing system.

**Design, Setting, and Participants:**

A cross-sectional study using a household sample was conducted in 10 areas of Gautam Budhnagar district, Uttar Pradesh, India, from January 31, 2020, to March 31, 2021, to assess the feasibility of identification of oral lesions by community health workers using a mobile phone application compared with diagnosis by trained dentists in a screening clinic. Men and women aged 30 years or older as well as tobacco users younger than 30 years were eligible for screening.

**Interventions:**

Screening by trained community health workers vs dentists.

**Results:**

A total of 1200 participants were screened by the community health workers during their home visits; of these, 1018 participants (526 [51.7%] men; mean [SD] age, 35 [16] years) were also referred and screened by the dentists a clinic. There was near-perfect agreement (κ = 0.9) between the findings of the community health workers and the dentists in identifying the positive or negative cases with overall sensitivity of 96.69% (95% CI, 94.15%-98.33%) and specificity of identification of 98.69% (95% CI, 97.52%-99.40%).

**Conclusions and Relevance:**

In this cross-sectional study, trained community health workers were able after initial supervision by qualified dentists to perform oral cancer screening programs. These findings suggest that community health workers can perform this screening in resource-constrained settings.

## Key Points

**Question:**

Is it feasible for community health workers to screen for oral cancer using a mobile application capturing system?

**Findings:**

In this cross-sectional study, 1200 participants were screened and the findings were compared with those of a subgroup who were also screened by dentists. There was near-perfect agreement between the findings of the community health workers and dentists in identifying positive or negative cases of oral lesions.

**Meaning:**

The findings of this study suggest that it is feasible to train community health workers to perform oral cancer screening in resource-limited settings.

## Teaser

This cross-sectional study examines the use of community health workers to perform screening of individuals for the presence of oral cancer in India.

## Introduction

The oral cancer burden in India is increasing, with 135 929 new cases and 75 290 deaths reported in 2020 and age-standardized incidence (9.8%) and mortality (5.4%) rates.^1^ India also contributes to one-third of the global oral cancer burden.^2^ Potentially malignant oral disorders include a variety of lesions and conditions characterized by an increased risk for malignant transformation.^3^ Screening may play an important role; for example, a large randomized clinical trial conducted more than 20 years ago in Kerala, India, showed a significant reduction in mortality.^4,5,6^ In this trial, nonmedical university graduates were trained to conduct oral visual inspection,^4^ and further research is warranted.^5,6^

The lower-cost, scalable option of community health worker–led oral cancer screening may be an important alternative.^7^ For example, accredited social health activists in India may be able to undertake these services; there is 1 accredited social health activist per 1000 residents who currently undertakes a variety of tasks including screening for noncommunicable diseases.^8^ Furthermore, community health workers contributed substantially to a policy program (Ayushman Bharat) that includes the National Programme for Prevention and Control of Cancer, Diabetes, Cardiovascular Diseases, and Stroke that is being gradually implemented nationally in India.^9^ Community health workers may be crucial in facing the growing challenge of health care access and delivery, but their role in low- and middle-income countries is less well studied.^10^

Earlier studies in which community health workers were provided a mobile phone with an application for oral cancer screening support the concept of integrating health and technology to improve public health surveillance.^7^ The electronic data captured have the advantage of maintenance of medical records, patient monitoring, and cloud-based storage service.^11^ However, there are few studies assessing the utility of community health workers in early oral cancer detection through a technology-based platform with follow-up and smoking counseling.^7^ Hence, the present study was undertaken to assess the feasibility of community health workers in screening and early detection of oral cancer using a mobile application capturing system.

## Methods

### Study Design and Setting

We conducted a cross-sectional analytical pilot study with a sample size of 1200 participants from 10 areas of Gautam Budhnagar district, Uttar Pradesh, India. Uttar Pradesh reported a tobacco use prevalence of 35.5% as per the Global Adult Tobacco Survey in 2015-2016, which is higher than the national average of 28.6% in India.^12^ Gautam Budhnagar district was chosen from Uttar Pradesh as the study area owing to its close proximity to our organization (Indian Council of Medical Research, National Institute of Cancer Prevention and Research, referred to as HPC from here on) for accessing the screening services by the participants with ease when referred. The study was performed from January 31, 2020, to March 31, 2021, and screening was conducted in the same period. Ethics approval for the study was obtained from the University of Warwick, Coventry, UK, and the Indian Council of Medical Research in India. Written and verbal informed consent were obtained in the local language (Hindi); participants did not receive financial compensation. This study followed the Strengthening the Reporting of Observational Studies in Epidemiology (STROBE) reporting guideline.

### Training of Community Health Workers

A 1-day hands-on training workshop was conducted at the HPC for the 10 selected community health workers to (1) systematically enumerate households and individuals using a household form; (2) interview the eligible individuals to elicit and record information on sociodemographic factors and tobacco and alcohol habits using the mobile application device; (3) perform a systematic visual inspection of the buccal and labial mucosa, gingivae, Buccoalveolar sulci, tongue, palate, and floor of the mouth under adequate lighting and using 2 disposable wooden spatulas; (4) identify any oral suspicious white and red lesions, trismus, and ulcers and growths suggestive of oral cancer, along with the referral criteria to the dental practitioner at our organization; and (5) photograph the oral cavity using the camera in the mobile phone.

These sessions were led by trained dentists from the organization (Indian Council of Medical Research) (V.T. and A.J.). The oral cancer screening application on the mobile phones of community health workers was used for training and data-capturing purposes. A training manual prepared by the faculty in the vernacular Hindi language for community health workers that provided descriptive and photographic documentation enabling identification of the different types of oral lesions was used as the resource material for the training.

A kit was provided to the community health workers containing tongue depressors, disposable masks, disposable gloves, flashlight, red medical waste bags, hand sanitizers, mobile phone for data entry, an easy-to-use reference manual for identification of oral lesions, and consent forms and referral cards in the local language. At the end of the training session, written tests were conducted to evaluate the level of understanding in identifying normal and abnormal lesions, use of the mobile application, and the knowledge gained by the community health workers. The top 5 ranked community health workers were first chosen to conduct screening and the remaining 5 community health workers received further training before deployment in the field. They were all paid for undertaking this extra screening activity.

### Study Population and Approach

All homes in the 10 areas were eligible for the screening program. As per the national guidelines for population cancer screening, all men and women aged 30 years or older, as well as tobacco users younger than 30 years, were eligible for screening.^13^

At the start of the program, a health awareness event was organized to encourage uptake. Then, the community health workers identified eligible individuals with the information provided in the household listing forms. Screening was done primarily by community health workers, with assistance from the dentists and a nurse during the initial days of the study. Specifically, the community health workers screened individuals using a mobile phone–based questionnaire (eAppendix 1 in the Supplement). During the first 3 months of the study, an average of 10 to 12 participants were screened per day and screening was stopped due to the COVID-19 lockdown in India from March 25 to May 31, 2020. Screening was gradually restarted in June 2020, with 5 to 6 participants screened per day with due consideration of adherence to COVID-19 guidelines. Capturing and uploading the photographs including normal mucosa and abnormal lesions in the oral cavity of the participants were undertaken using the oral cancer screening mobile application in the provided android phone (Redmi Note 9; Xiaomi Inc). The images were captured in autofocus and auto flash modes. If the community health worker noted the presence of any oral potentially malignant disorders (eg, white, red, or mixed lesions; ulcers; and growth masses), apparently normal mucosa, and apparently normal mucosa, they informed the participant. Yellow referral cards were provided to the participants with normal findings. Red cards were provided to participants with suspected oral lesions, and they were encouraged to visit the HPC for further examination by the dentists and recording of appropriate details in the online clinic (eAppendix 2 in the Supplement). The participants were provided with the appropriate diagnosis, and the photographs of the oral cavity were taken with a high-resolution digital single-lens reflex camera (Nikon D5600; Nikon Inc) by the dentists. Punch biopsies were performed on suspicious lesions by trained dentists and reported as per World Health Organization guidelines (ie, hyperkeratosis; mild, moderate, or severe dysplasia; carcinoma in situ; and squamous cell carcinoma).^13^ Individuals diagnosed with squamous cell carcinoma were referred to a tertiary care center for further investigation and appropriate management.

All participants were informed of the ill effects of tobacco and the importance of routine oral self-examination. They were advised to undergo screening every 5 years per the national guidelines or whenever they noticed any abnormality in their oral cavity.^13^ In addition, smokers were referred to the tobacco cessation clinic. Individuals who were tobacco users and had oral potentially malignant disorders underwent tobacco counseling in the tobacco cessation clinic. They were offered 4 to 8 counseling sessions lasting 30 to 60 minutes. Patients with potentially malignant oral disorders were followed up for up to 3 months depending on the size and site of the lesion, smoking cessation, presence of underlying disease, and change in the size of the lesion. Reasons for nonparticipation were also noted.

### Statistical Analysis

The data collected in the oral cancer screening mobile application were exported to a Microsoft Excel 2018 (Microsoft Corp) spreadsheet and analyzed using Stata, version 14 (StataCorp LLC). Sensitivity, specificity, and positive and negative predictive values were calculated. The interrater agreement was analyzed with the Cohen κ coefficient test. With paired, 2-sided testing, the significance threshold was *P* < .001.

## Results

A total of 1200 individuals were approached by community health workers for screening with 98% uptake. Initially, 10 to 12 participants were screened per day, but this stopped as India went into lockdown (March-May 2020) due to the COVID-19 pandemic. The main reasons for nonparticipation (total, 182; 54% men and 48% women) included the perception of being healthy and lack of time or interest.

Of these, 1018 participants (526 [51.7%] men and 492 [48.3%] women) visited HPC for screening by the dentists (Table 1). Of the total population screened, most were aged 30 to 39 years (338 [33.2%]), and the mean (SD) age of participants was 35 (16) years. Smokeless tobacco (35%; 95% CI, 33.5%-36.5%) was consumed more commonly than smoked tobacco (35%; 95% CI, 32.9-37.0) products in both sexes, with a high predilection of both tobacco and alcohol use among men.

**Table 1.** Substance Use History Among the Population Screened by Community Health Workers and Dentists

Substance use	Sex	Age group, No. (%), y							Total
		0-19	20-29	30-39	40-49	50-59	60-69	≥70	
No. (%)		17	175	338	324	99	53	12	1018
No use	Male	1 (1.5)	12 (17.6)	21 (17.6)	27 (39.7)	3 (4.4)	4 (3.8)	0	68 (6.7)
	Female	0	9 (2.7)	150 (44.9)	133 (39.8	25 (7.5)	14 (4.2)	3 (0.9)	334 (32.8)
Smoking only	Male	6 (2.7)	63 (28.8)	52 (23.7)	50 (22.8)	26 (11.9)	17 (7.8)	5 (2.3)	219 (21.5)
	Female	0	4 (19)	0	7 (33.3)	7 (33.3)	3 (14.2)	0	21 (2.1)
Smokeless tobacco (SLT) users	Male	13 (3.8)	81 (23.8)	103 (30.2)	98 (28.7)	32 (9.4)	13 (3.8)	1 (0.3)	341 (33.5)
	Female	1 (0.8)	33 (26)	34 (26.8)	39 (30.7)	10 (5.5)	7 (5.5)	3 (2.4)	127 (12.5)
Areca nut^a^	Male	1 (3.1)	6 (18.8)	9 (28.1)	11 (34.3)	2 (6.3)	3 (9.4)	0	32 (3.1)
	Female	0	3 (16.7)	10 (55.6)	3 (16.7)	1 (5.6)	1 (5.6)	0	18 (1.8)
Dual use (smoking and SLT users)	Male	4 (3.5)	29 (25.4)	33 (28.9)	33 (28.9)	8 (7)	7 (6.1)	0	114 (11.4)
	Female	0	1 (33.3)	0	2 (66.7)	0	0	0	3 (0.3)
Smoking and alcohol	Male	3 (3.1)	27 (27.8)	25 (25.8)	25 (25.8)	12 (12.4)	4 (4.1)	1 (1)	97 (9.7)
	Female	0	0	0	0	1 (100)	0	0	1 (0.09)
Smokeless tobacco use and alcohol users	Male	5 (3.6)	38 (27.5)	44 (31.9)	36 (26.1)	10 (7.2)	5 (3.6)	0	138 (13.6)
	Female	0	0	0	3 (100)	0	0	0	3 (0.3)

^a^ Areca nut, commonly known as betel nut.

Table 2 reports the oral visual examination findings referred by the community health workers and screened by the dentists. White patch lesions (leukoplakia) were the most common lesion in both sexes (148 [14.5%]), followed by oral submucous fibrosis (79 [7.8%]) and tobacco pouch keratosis (81 [8.0%]).

**Table 2.** Findings on Participants Referred to Dentists

Visual findings	No. (%)		
	Men	Women	Total
No.	526 (51.7)	492 (48.3)	1018
White patch lesions			
Leukoplakia	129 (24.5)	19 (3.9)	148 (14.5)
Oral lichen planus	4 (0.8)	5 (1.0)	9 (0.9)
Red patch lesions			
Erthyroplakia	1 (0.2)	0	1 (0.09)
Ulcer			
Recurrent aphthous ulcer	30 (5.7)	18 (3.7)	48 (4.7)
Growth mass			
Malignant	23 (4.4)	4 (0.8)	27 (2.7)
Other			
Oral submucous fibrosis	57 (10.8)	22 (4.5)	79 (7.8)
Tobacco pouch keratosis	74 (14.1)	7 (1.4)	81 (8.0)
Lichenoid lesion	2 (0.4)	4 (0.8)	6 (0.6)
Mixed lesions			
Leukoplakia + tobacco pouch keratosis	2 (0.4)	0	2 (0.2)
Oral submucous fibrosis + tobacco pouch keratosis	2 (0.4)	0	2 (0.2)
Normal	202 (38.4)	413 (83.9)	615 (60.4)

### Reliability in Identification by Community Health Workers

The reproducibility of community health workers’ examinations and their agreement with the dentists’ outcomes was measured using κ statistics.^14^ The sensitivity and specificity of the oral visual examination by the community health workers were calculated by pooling all the results as well as by stratification according to participant sex and age.

The interrater agreement of the oral visual examination findings among the 3 dentists at our organization was done in 100 participants and showed near-perfect agreement between them as indicated by the overall κ value of 0.9 (*P* < .001). There also was near-perfect agreement between the findings of the 3 dentists as indicated by the overall κ value of 0.9 (*P* < .001).

eTable 1 in the Supplement reports the identification of oral lesions by community health workers and dentists in the screened population: there was near-perfect agreement between the dentists and the community health workers in designating participants with positive or negative cases (κ = 0.9; *P* < .001). The overall sensitivity of the total findings by both dentists and community health workers was 96.69% (95% CI, 94.15%-98.33%) and the specificity of identification was 98.69% (95% CI, 97.52%-99.40%). The positive likelihood ratio was 73.70%, and the negative likelihood ratio of identification of oral lesions was 0.03%. The prevalence of disease was 32.41% with a positive predictive value of 97.20% and negative predictive value of 98.44%, with an accuracy of 98.29%.

Of the 1018 participants screened by the dentists, 686 (67.4%) were designated as having no potentially malignant oral disorders and 332 (32.6%) participants were diagnosed with potentially malignant oral disorders. Leukoplakia (n = 7), oral submucous fibrosis (n = 2), and recurrent aphthous ulcers (n = 2) were misdiagnosed as normal (ie, false-negative) by the community health workers. The screening undertaken by community health workers showed that 688 participants (67.6%) had no abnormalities and 330 (32.4%) had a potentially malignant oral disorder. A total of 27 oral cancers (2.7%) were detected in the study.

eTable 2 in the Supplement reports the concordance between the results of the oral visual examination by community health workers and the dentists based on the type of lesion. When considering single types of lesions, the agreement was also very good, as evidenced by the κ statistics, with κ = 1.0 indicating perfect agreement for the red patch lesions (erythroplakia) and growth mass (malignant neoplasm); κ  = 0.9 indicating near-perfect agreement for the white patch lesions, such as leukoplakia and oral lichen planus; and κ  = 0.9 indicating near-perfect agreement for the ulcers and others types of lesions, such as oral submucous fibrosis, tobacco pouch keratosis, and lichenoid lesions. The overall agreement between the community health workers and the dentists in identifying the different types of lesions was high (κ = 0.9).

In a 3-month follow-up period, potentially malignant oral disorders, such as homogeneous leukoplakia, showed regression or complete resolution in 22 patients (17 men, 5 women) concurrent with tobacco cessation, management, and follow-up provided by the dentists. Twenty-six participants either stopped or reduced tobacco use.

## Discussion

Our results help expand the literature on screening for oral cancer by showing the feasibility of community health workers performing screenings with mobile cameras with high accuracy. Our findings that the sensitivity of oral cancer screening visually by community health workers was 96.6%, and specificity was 98.6% when referred to the dentists compared favorably with those in a study reported by Mathew et al^15^ in which community health workers undertook screening. Other studies have found that the sensitivity of oral cavity inspection visually varied from 57.1% to 61.4%, and the specificity ranged from 98.6% to 98.8%.^4,16,17^

A study done in Ernakulam district, Kerala, India, to assess the use of community health workers in performing the oral visual inspection showed a lower sensitivity of 56% and comparable specificity of 98% compared with our study.^18^ In an earlier Sri Lankan study, 29 215 (33.5%) of the 87 277 eligible participants aged 20 years and older were examined by community health workers.^19^ Some 565 of the 1220 referred participants (46.3%) were further examined by dentists in which 338 had white patch lesions (59.8%), 14 had malignant lesions (2.4%), and 213 had benign or no lesions (37.6%). Almost similar results were obtained when this model was reproduced in another region of Sri Lanka.^20^ In contrast, a long-term feasibility study to evaluate the role of community health workers in the early detection of oral cancer in Trivandrum district, Kerala, India, did not motivate the community health workers, and 90% of them did not participate in the program.^21^

During 1984-1990, dentists in the Cuban health services provided the screening test in which 12 990 677 examinations were performed and 30 244 participants (0.23%) were referred to hospitals. Among them, 8.1% had oral cancer, 37% oral potentially malignant disorders, and 54.9% had either benign or no lesions. Thus, the false-positive referrals in the reported studies of oral cancer screening varied from 20% to 55%.^22^ Community health workers have also played roles in previous studies to address the feasibility of using health care auxiliary personnel in the control of oral cancer in India and Sri Lanka.^18,19,20^ These studies suggest that trained community health workers can undertake a systematic mouth examination and identify lesions.

Although oral cancer and oral visual examination meet most of the criteria of Wilson and Jungner^23^ for a suitable disease and screening test, respectively, there are few randomized clinical trials to support the usefulness of screening for oral cancer as being effective in reducing the incidence and mortality associated with this disease.^24^ Sankaranarayanan et al^4^ and Mathew et al^21^ have reported that nonmedical graduates can identify oral lesions by visual screening and their role in oral cancer screening programs. They suggested that oral screening of high-risk individuals be established in routine health services in India, given the high disease burden. However, caution is required because there are methodologic weaknesses in these and other studies, including no analysis of clustering, blinding of outcome, and loss to follow-up.^15,16,24^

In our study, the agreement between the results of oral examination by community health workers and the dentists was notable because, as the number of participants recruited in the study increased, the community health workers accumulated further experience in identifying oral mucosal lesions. Also, the retraining provided by the dentists along with additional monetary incentives improved the performance of the community health workers in screening and referrals of participants by them to our organization.

Our study builds on the work of Braun et al^25^ and Källander et al,^26^ showing that community health workers have used mobile technology in house-to-house screening data collection, promoting health education, counseling, and referral for further care in various domains of health. A study by Birur et al^27^ noted the novelty of the mobile phone health–based approach and aided remote early detection of oral cancer by community health workers in a resource-constrained setting. In our study, community health workers were trained to capture the data electronically and obtain photographs of the oral mucosa using android phones. The use of mobile phone technology for data collection and image capturing in the offline mode was a new experience for the community health workers and we faced a few challenges during the screening process, such as capturing images of an inappropriate subsite of the oral cavity, missing imaging of the lesion, and poor-quality images. These shortcomings occurred during the initial part of the study; with the passage of time and increasing experience in handling the devices, these issues were resolved.

### Strengths and Limitations

Strengths of our study suggest that community health workers can undertake oral screening. We were able to follow up participants with potentially malignant oral disorders and, in 22 participants, there was a decrease in the size of the lesion and, through tobacco cessation counseling, 26 participants stopped this habit. Furthermore, electronic data collection using android mobile phones with data transfer when connected to the internet increased data accessibility for researchers. The high-quality oral images of the participants screened by dentists may serve as a repository for further researchers. None of the community health workers dropped out of the oral cancer screening in the community program throughout the duration of our study.

The study also has limitations. The intraobserver agreement among 10 community health workers in identifying normal and abnormal oral lesions was not calculated and the study was not powered to detect differences. In addition, participants were selected from a single geographic location, so the findings cannot be generalized.

The 9 potentially malignant oral disorders and 2 recurrent aphthous ulcers missed by the community health workers were present in the lower vestibular mucosa, buccal mucosa, and lower lip. This lack of identification indicates that these areas in the oral cavity may have been inaccessible owing to improper retraction.

## Conclusions

The findings of this study suggest feasibility of training community health workers to perform oral cancer screening in a low- or middle-income country. However, a randomized clinical trial is needed to determine the effectiveness and cost-effectiveness of this approach.

## Notes

Supplement. **eAppendix 1.** Mobile-Based Questionnaire by CHW **eAppendix 2.** Details Collected by the Dentists at ICMR-NICPR-HPC **eTable 1.** Identification of the Oral Lesion by CHWs and Dentists in the Screened Population **eTable 2.** Distribution and Agreement by Type of Individual Findings on Oral Visual Inspection by CHWs and Dentists
