# Supplementary material for: Feasibility of Training Community Health Workers in the Detection of Oral Cancer
Source: JAMA Netw Open. 2022 Jan 18;5(1):e2144022. doi: 10.1001/jamanetworkopen.2021.44022 (PMC8767429; doi:10.1001/jamanetworkopen.2021.44022)
Supplement: Supplement. — eAppendix 1. Mobile-Based Questionnaire by CHW eAppendix 2. Details Collected by the Dentists at ICMR-NICPR-HPC eTable 1. Identification of the Oral Lesion by CHWs and Dentists in the Screened Population eTable 2. Distribution and Agreement by Type of Individual Findings on Oral Visual Inspection by CHWs and Dentists [file jamanetwopen-e2144022-s001.pdf]

Supplementary Online Content

Thampi V, Hariprasad R, John A, et al. Feasibility of training community health workers in the detection of oral cancer. *JAMA Netw Open*. 2022;5(1):e2144022. doi:10.1001/jamanetworkopen.2021.44022

eAppendix 1. Mobile-Based Questionnaire by CHW

eAppendix 2. Details Collected by the Dentists at ICMR-NICPR-HPC

eTable 1. Identification of the Oral Lesion by CHWs and Dentists in the Screened Population

eTable 2. Distribution and Agreement by Type of Individual Findings on Oral Visual Inspection by CHWs and Dentists

This supplementary material has been provided by the authors to give readers additional information about their work.

© 2022 Thampi V et al. *JAMA Network Open.*

eAppendix 1: Mobile-based questionnaire by CHW

1.Name of the participant
2.Age of the participant
3.Select participant's gender
4.Address of the participant
5.Contact no
6.Alternative contact no
7.Tobacco chewer
 a. Yes b. No
8.Smoker
 a. Yes b. No
9.Alcohol
 a. Yes b. No
10.Arecanut
 a. Yes b. No
11.Does the participant had any symptoms of mouth ulcer, bleeding from the teeth or gums, difficulty in swallowing, pain or burning sensation, decreased mouth opening or others: Yes, or No
12.On Oral visual examination by the CHW did the participant had any white patch, red patch, growth mass, ulcer, others: Yes, or No

eAppendix 2: Details collected by the dentists at ICMR-NICPR-HPC

1.Date of registration
2.Patient's name
3.Age/Sex
4.ID no
5.Mobile no
6.Smoking: cigarette/bidi/hookah-times per day, years, quit since
7.Chewing raw tobacco: times per day, years, quit since
8.Gutkha: times per day, years, quit since
9.Pan masala: times per day, years, quit since
10.Arecanut: times per day, years, quit since
11.Alcohol intake: times per day, years, quit since
12.Site of the lesion:
13.Provisional diagnosis:
14.Type of investigation:
15.Site of investigation:
16.Date of investigation:
17.Result of investigation:
18.Type of treatment given:
19.Recall:

© 2022 Thampi V et al. *JAMA Network Open.*

eTable 1. Identification of the oral lesion by CHWs and dentists in the screened population

TOTAL		Dentists' findings Positive	Negative	Total
CHW findings	Positive	321	9	330
	Negative	11	677	688
	Total	332	686	1018

Statistic	Value (95% CI)
Sensitivity	96.69% (94.15 to 98.33%)
Specificity	98.69% (97.52 to 99.40%)
Positive Likelihood Ratio	73.70 (38.50 to 141.08)
Negative Likelihood Ratio	0.03 (0.02 to 0.06)
Disease prevalence (*)	32.41%
Positive Predictive Value (*)	97.20% (94.77 to 98.52%)
Negative Predictive Value (*)	98.44% (97.25 to 99.12%)
Accuracy (*)	98.29% (97.29 to 98.99%)

(*) These values are dependent on disease prevalence

© 2022 Thampi V et al. *JAMA Network Open.*

eTable 2. Distribution and agreement by type of individual findings on oral visual inspection by CHWs and dentists

CHW findings		Dentists findings			
		Positive	Negative	Total	Kappa
**White patch**	Positive	150	6	156	
	Negative	7	855	862	
	Total	157	912	1018	**0.9**
**Red patch**	Positive	1	2	3	
	Negative	0	1015	1015	
	Total	1	1017	1018	**1.0**
**Growth mass**	Positive	27	2	29	
	Negative	0	989	989	
	Total	27	991	1018	**1.0**
**Ulcer**	Positive	46	2	48	
	Negative	2	968	970	
	Total	48	970	1018	**0.9**
**Others**	Positive	180	1	181	
	Negative	2	825	827	
	Total	182	826	1018	**0.9**
